# PLZF-RAR_α_, NPM1-RAR_α_, and Other Acute Promyelocytic Leukemia Variants: The PETHEMA Registry Experience and Systematic Literature Review

**DOI:** 10.3390/cancers12051313

**Published:** 2020-05-21

**Authors:** Marta Sobas, Maria Carme Talarn-Forcadell, David Martínez-Cuadrón, Lourdes Escoda, María J. García-Pérez, Jose Mariz, María J. Mela-Osorio, Isolda Fernández, Juan M. Alonso-Domínguez, Javier Cornago-Navascués, Gabriela Rodríguez-Macias, María E. Amutio, Carlos Rodríguez-Medina, Jordi Esteve, Agnieszka Sokół, Thais Murciano-Carrillo, María J. Calasanz, Manuel Barrios, Eva Barragán, Miguel A. Sanz, Pau Montesinos

**Affiliations:** 1 Blood Neoplasms and Bone Marrow Transplantation, Department of Hematology, Wroclaw Medical University, 50-367 Wrocław, Poland; marta.sobas@umed.wroc.pl; 2 Hospital of Tarragona “Joan XXIII”, Hematology-ICO, 43-005 Tarragona, Spain; ctalarn@iconcologia.net (M.C.T.-F.); lescoda@iconcologia.net (L.E.); 3 Department of Hematology, Hospital Universitari I Politècnic La Fe, 46-009 Valencia, Spain; martinez_davcua@gva.es (D.M.-C.); Miguel.Sanz@uv.es (M.A.S.); 4 CIBERONC Instituto de Salud Carlos III, 28-020 Madrid, Spain; barragan_eva@gva.es; 5 Department of Hematology, University Hospital Torrecardenas, 04-009 Almeria, Spain; mj.garciaper@gmail.com; 6 Department of Hematology, Istituto Portugues de Oncologi IPO, 4200-072 Porto, Portugal; mariz@ipoporto.min-saude.pt; 7 Fundaleu, Department of Hematology, Buenos Aires 1114, Argentina; mjmela@fundaleu.org.ar (M.J.M.-O.); ifernandez@fundaleu.org.ar (I.F.); 8 Department of Hematology, University Hospital Universitario Fundacion Jimenez Diaz IIS-FJD, 28-040 Madrid, Spain; juan.adominguez@fjd.es (J.M.A.-D.); javier.cornago@hospitalreyjuancarlos.es (J.C.-N.); 9 Deapartment of Hematology, Hospital Gregorio Maranion, 28-010 Madrid, Spain; mgabrielarm@yahoo.com; 10 Department of Hematology, Hospital de Cruces, 48-903 Barakaldo, Spain; mariaelena.amutiodiez@osakidetza.net; 11 Department of Hematology, Hospital Universitario Dr. Negrin, 35-010 Las Palmas de Gran Canaria, Spain; hematocritico@yahoo.es; 12 Department of Hematology, Hospital Clinic, 08-036 Barcelona, Spain; jesteve@clinic.cat; 13 Department of Paediatric Bone Marrow Transplantation, Oncology and Hematology, Wroclaw Medical University, 50-367 Wrocław, Poland; agnieszkasokoljezewska@interia.pl; 14 Department of Pediatric Hematology, Hospital Vall d’Hebron, 08-035 Barcelona, Spain; thais.murciano@vhebron.net; 15 Department of Hematology, Clinica Universitaria de Navarra, 31-008 Pamplona, Spain; mjcal@unav.es; 16 Department of Hematology, Hospital Carlos Haya, 29-014 Málaga, Spain; barriosmanolo1@gmail.com; 17 Department of Molecular Biology Laboratory, Hospital Universitari I Politècnic La Fe, 46-009 Valencia, Spain

**Keywords:** variant, acute promyelocytic leukemia, systematic review, characteristics, outcomes

## Abstract

It has been suggested that 1–2% of acute promyelocytic leukemia (APL) patients present variant rearrangements of retinoic acid receptor alpha (RARα) fusion gene, with the promyelocytic leukaemia zinc finger (PLZF)/RAR_α_ being the most frequent. Resistance to all-trans-retinoic acid (ATRA) and arsenic trioxide (ATO) has been suggested in PLZF/RAR_α_ and other variant APLs. Herein, we analyze the incidence, characteristics, and outcomes of variant APLs reported to the multinational PETHEMA (Programa para el Tratamiento de Hemopatias Malignas) registry, and we perform a systematic review in order to shed light on strategies to improve management of these extremely rare diseases. Of 2895 patients with genetically confirmed APL in the PETHEMA registry, 11 had variant APL (0.4%) (9 PLZF-RAR_α_ and 2 NPM1-RAR_α_), 9 were men, with median age of 44.6 years (3 months to 76 years), median leucocytes (WBC) 16.8 × 10^9^/L, and frequent coagulopathy. Eight patients were treated with ATRA plus chemotherapy-based regimens, and 3 with chemotherapy-based. As compared to previous reports, complete remission and survival was slightly better in our cohort, with 73% complete remission (CR) and 73% survival despite a high relapse rate (43%). After analyzing our series and performing a comprehensive and critical review of the literature, strong recommendations on appropriate management of variant APL are not possible due to the low number and heterogeneity of patients reported so far.

## 1. Introduction

Acute promyelocytic leukemia (APL) is a relatively rare hematologic malignancy accounting for ~10% of acute myeloid leukemia (AML) cases [1]. APL is characterized by M3 morphological subclassification, t(15;17)(q22;q21) chromosomal translocation, and promyelocytic leukemia protein (PML)—retinoic acid receptor alpha (RAR_α_) gene fusion, showing high rates of complete remission (CR) and cure using front-line schedules with all-trans-retinoic acid (ATRA) and/or arsenic trioxide (ATO). Apart from the classical PML-RAR_α_ cases, some APL patients are diagnosed with rare APL variants, characterized by a different rearrangement involving RAR_α_ plus another partner gene. According to some reviews, these APL genetic variants account for ~1–2% of APL cases, but the real frequency remains unknown [2,3,4,5]. To our knowledge, 14 types of variant APL where RAR_α_ is fused to different genes have been reported [2,3,4,5,6,7,8,9,10,11,12,13,14,15,16,17,18,19,20,21,22,23,24,25,26,27,28,29,30,31,32,33,34,35,36,37,38,39,40,41,42,43,44,45,46,47,48,49,50,51,52,53,54,55,56,57,58,59,60,61,62,63]. Moreover, the clinical features and optimal treatment of variant APLs remain to be established. In fact, some authors have suggested resistance to ATO and ATRA for the PLZF-RAR_α_ APL, which is probably the most frequent form of rare APL forms (up to 0.8% of all APLs) [3,27].

In this study, we aim to analyze the incidence, characteristics, treatment patterns, and outcomes of variant APLs reported to the PETHEMA epidemiology registry, including patients from Poland, Argentina, Portugal, and Spain from PALG (Polish Adult Leukemia Group), PETHEMA (Programa para el Tratamiento de Hemopatias Malignas), and GATLA (el Grupo Argentino de Tratamiento de la Leucemia Aguda) groups. Given the clinical heterogeneity and scarce number of variant APL patients reported in the literature, we also aim to perform a systematic review in order to shed light on strategies to improve the management of these extremely rare diseases.

## 2. Methods

### 2.1. Patients and Eligibility

Between November 1996 and January 2020, adult patients from institutions from Spain, Poland, Portugal, Argentina, Colombia, and Uruguay were registered in the PETHEMA database. In all patients, the diagnosis of APL was suspected by cytomorphology and confirmed by conventional cytogenetics and/or reverse transcriptase-polymerase chain reaction (RT-PCR). Patients were reported irrespectively of the treatment administered, including also those dying early before starting ATRA or chemotherapy. Eligibility criteria and trial design for PML-RAR_α_ patients have been reported elsewhere [64,65,66,67]. Patients diagnosed with rare APL variants were not eligible as per PETHEMA trial protocols, but baseline and treatment information were also collected using the same forms than for PML-RAR_α_ APLs. Diagnosis of APL variants was performed after clinical suspicion (M3 morphology and lack of genetic diagnosis of t(15;17) or PML-RAR_α_). Specific PCR tests for rare rearrangements involving RAR_α_ were performed locally according to routine clinical practice. Two central laboratories were available in Spain as per physician’s demands in case of need. Informed consent was obtained from patients. According to the Declaration of Helsinki, the protocol was approved by the Research Ethics Board of each participating hospital.

### 2.2. Treatment

No specific guidelines or recommendations for front-line or salvage therapy for rare APL variants were available during the study period. If they considered appropriate, physicians could follow front-line protocols for t(15;17) APL, where induction therapy consisted of oral ATRA and intravenous idarubicin (AIDA regimen). All patients in complete remission (CR) received 3 anthracycline-based consolidation courses, which were risk-adapted since protocol LPA99. Consolidation was followed by 2 years of maintenance, as it was previously described [64,65,66,67]. Since 2017, the PETHEMA guidelines recommended ATO+ATRA combination for low- and intermediate-risk t(15;17) APL.

### 2.3. Response Assessment

Remission induction response and relapse were assessed according to the revised criteria by Cheson et al. [68]. There were no guidelines or definitions for molecular remission, molecular persistence, or molecular relapse. Relapse was defined as presence of equal or more than 5% bone marrow blasts or presence of extramedullary disease. Lumbar puncture or other diagnostic tests to assess disease status were performed as per physician’s judgement.

### 2.4. Data Collection

Data were collected and registered prospectively; last patient follow-up was updated on 15 February 2020. Following data were collected at diagnosis: age, gender, ECOG score, thrombosis and bleeding, hemoglobin level, platelet count, leukocytes (WBC) count, creatinine, uric acid, urea, lactate dehydrogenase (LDH), alkaline phosphatases, total bilirubin, and albumin, triglycerides, cholesterol, bilirubin, and type of RAR_α_ rearrangement. Coagulopathy was defined as thrombocytopenia plus either: prolonged prothrombin time and/or activated partial thromboplastin; or hypofibrinogenemia and/or increased levels of fibrin degradation products or D-dimers.

### 2.5. Systematic Review: Search Strategy and Selection of Studies

In accordance with the Preferred Reporting Items for Systematic Reviews and Meta-Analyses (PRISMA) guidelines, two independent reviewers (M.S. and P.M.) conducted this systematic review [69]. The following databases were searched without restrictions: PubMed and Excerpta Medica database (EMBASE). In addition, the reference lists of important studies and reviews were hand searched. The last literature search was performed on 23 February, 2020. Similar keywords were used in different databases: acute promyelocytic leukemia and “rare” or “variant” [Mesh], as well as the specific fusion genes. Case reports or studies analyzing a series of patients with rare APL variants were selected. Our systematic search obtained 60 citations from databases and journals (58 original research/case report articles and 2 reviews). The agreement in the study selection between the reviewers was excellent (kappa = 0.97).

## 3. Results

### 3.1. Patients and Eligibility

Overall, 2961 patients with newly diagnosed APL from Spain, Portugal, Argentina (GATLA group), Uruguay, Colombia, and Poland (PALG group) were reported to the PETHEMA registry. Of them, 66 were coded as lack of genetic diagnosis, resulting in 2895 patients with genetically confirmed APL. Among those, 11 patients with variant APL were identified (0.4%): 9 PLZF-RAR_α_ (0.3%) and 2 NPM1-RAR_α_ (0.1%). The main characteristics of variant APL patients are presented in Table 1. Briefly, the majority were men (9/11), with median age at diagnosis of 44.6 years (3 months to 76 years) in PLZF-RAR_α_ and 7 and 74 years for NPM1-RAR_α_ patients (two pediatric patients overall). Median WBC at diagnosis was 16.8 × 10^9^/L, with 50% patients classified as high-risk according to Sanz’s relapse-risk score [10,11,13]. Three out of 10 patients (30%) had hyperleukocytic forms with WBC > 50 × 10^9^/L. Two out of 11 patients had therapy-related APL secondary to colon neoplasm’s chemotherapy or to methotrexate plus antipaludic drugs for rheumatoid arthritis. The majority of variant APL had coagulopathy at diagnosis (6 out of 7 with available data), and 2 out of 6 patients with CD56 blast expression available were positive. 

### 3.2. Treatment and Outcomes

All patients received induction treatment, consisting of ATRA plus idarubicine (IDA) according to the ongoing PETHEMA trial (AIDA regimen) in 5 patients, AML-type chemotherapy plus ATRA in 3 patients (1 patient IDICE, 1 patient 3 + 7, and 1 patient AIE) (Table 1). Three remaining patients received an AML-type chemotherapy without ATRA or ATO (2 patients 3 + 7, and 1 patient FLUGA). CR was achieved in 8 patients (73%), with 1 resistance and 2 induction deaths (18%) (76 and 43 years old, both treated with AIDA and with WBC > 10 × 10^9^/L and coagulopathy at presentation). The initially resistant patient received second-line regimen with AIDA (partial remission) and reached a first CR after a 2 + 5 regimen (Ida + Ara-C).

Post-remission schedule consisted of ATRA and anthracycline-based consolidation cycles in 4 patients (3 of them followed by ATRA plus low-dose chemotherapy maintenance) and intermediate or high dose Ara-C-based (HD-Ara-C) consolidation in 5 patients. Two patients (40 and 50 years old) received an allogeneic hematopoietic stem cell transplant (alloHSCT) in first CR. Only one patient (0.3 years old) received central nervous system (CNS) prophylaxis as part of the front-line regimen.

With a median follow-up of 60 months (8–101 months), 8 patients (73%) were alive at the time of analysis (3 deaths: 2 induction deaths and 1 death due to disease progression after third relapse). Overall, 4 out of 9 patients (43%) achieving first CR subsequently relapsed at 9, 24, 40, and 58 months (3 of them had CNS relapse). Patients relapsing had 53, 100, and 248 × 10^9^/L WBC counts at diagnosis (1 patient no available data). A second CR was achieved in all cases (salvage therapy was ATRA + ATO in 3 patients; and HD-Ara-C in 1; including triple intrathecal chemotherapy for all patients with CNS relapse). An alloHSCT was performed in second CR in 1 patient who relapsed subsequently, and 1 patient was planned to undergo Haploidentical alloHSCT.

### 3.3. Systematic Literature Review

#### 3.3.1. PLZF-RAR_α_ APL

Chen et al. described in 1993 the first variant APL with t(11;17)(q23;q21), producing the promyelocytic leukemia zinc finger PLZF-RAR_α_ fusion gene [61] (also known as ZBTB16-RAR_α_). In a multicenter study performed by Grimwade et al., 5 out of 611 APL patients (0.8%) had the PLZF-RAR_α_ fusion gene being so far the only source to estimate the frequency of variant APLs [3]. PLZF-RAR_α_ APL seems to have distinct morphological features, as compared with classical APL, being the blast nucleus regular vs. bilobed, granules are fine or absent, and there is an increased CD56 expression by flow cytometry (similar to atypical FAB M3v APL) [4,11,66].

To date, 35 cases of PLZF-RAR_α_ APL have been reported (Table 2). Briefly, the vast majority were men (32/35) with median age at diagnosis of 48 years (15–81 years) and median WBC 9.9 × 10^9^/L, with 14 out of 34 (41%) patients classified as high-risk according to the Sanz’s relapse-risk score. Five out of 35 patients (15%) had WBC > 50 × 10^9^/L. All patients received first induction therapy that resulted in 12 CR (34%), 18 resistances (51%), and 5 induction deaths (14%). Five patients received ATRA monotherapy (1 resistance and 4 induction deaths), 14 ATRA plus chemotherapy-based regimens (9 CR [64%], 4 resistances, and 1 induction death), 6 AML-type chemotherapy (2 CR and 4 resistances), 6 ATO+/-chemotherapy-based induction (6 resistances), and 4 ATO+ATRA+/-chemotherapy-based induction (1 CR and 3 resistances). Accounting for first-line and salvage induction therapy, 23 patients achieved a first CR (66%). Successful salvage induction consisted of AML-type chemotherapy in 7, ATRA plus chemotherapy in 2, ATRA monotherapy in 1, and ATO+ATRA in 1 patient. Overall, 26 patients were exposed to ATRA at any therapeutic line (CR in 14, resistance in 9 [3 of them with signs of differentiation], and 3 were non-evaluable because of early death) and 12 were exposed to ATO (CR in 2 and resistance in 10). Two out of 23 patients achieving a first CR received subsequently an alloHSCT. Regarding survival, seven patients were not evaluable (Table 2). The median follow-up of patients alive was 17 months (21–77 months), with 14 patients (48%) alive (15 deaths: 5 induction deaths, 2 deaths without relapse, and 8 deaths after relapse). Overall, 12 out of 21 patients (57%) relapsed after first CR (no CNS relapse reported), and 4 patients received an alloHSCT in first or second CR [3,4,5,6,8,9,10,11,12,13,14,24,25,26,28,29,30,31,32,33,34,63].

#### 3.3.2. NPM1-RAR_α_ APL

We identified 9 cases of NPM1-RAR_α_ (Table 3), with median age at diagnosis of 12 years (2–76 years). Overall, 5 (55.6%) patients were pediatric, the majority were male (6 patients), median WBC was 15.7 × 10^9^/L, and 3 had extramedullary disease. All patients received first induction therapy that resulted in 7 CR (78%) and 2 induction deaths (22%). One patient received ATRA monotherapy (1 induction death), 6 received ATRA plus chemotherapy-based regimens (5 CR and 1 induction death), and 2 an AML-type chemotherapy (2 CR). No clinical data regarding ATO sensitivity were reported. No patients achieving a first CR received an alloHSCT. The median follow-up of patients alive was 23 months (2–46 months), with 6 patients (67%) alive (2 induction deaths, and 1 death after relapse). Overall, 4 out of 7 patients (57%) relapsed after first CR (no CNS relapse reported). One patient received an alloHSCT in second CR [3,6,35,36,37,38,39,40,41].

#### 3.3.3. Other Variant APL

Overall, 35 additional APL patients with other RAR_α_ fusion genes have been reported as follow: 12 with the signal transducer and activator of transcription 5 beta (STAT5B)-RARα, 6 with Transducin β-like 1 X-linked receptor 1 (TBLR1-RARα), 5 with interferon regulatory factor 2 binding proteins 2 (IRF2P2)-RARα, 2 with factor interacting with PAPOLA and CPSF1 (FIP1L1)-RAR_α_, 2 with signal transducer and activator of transcription 3 (STAT3)-RAR_α_, 2 with BCL6 Corepressor (BCOR)-RAR_α_, and 1 case of nuclear mitotic apparatus protein 1(NuMa1)-RARα, protein Kinase CAMP-Dependent Type I Regulatory Subunit Alpha (PRKAR1A)-RARα, nucleic acid binding protein 1 (NABP1)-RARα, general transcription factor II-I (GTF2I)-RARα, trafficking From ER To Golgi Regulator (TFG)-RARα, and fibronectin Type III Domain Containing 3B (FNDC3B)-RARα (Table 4) [7,15,16,17,18,19,20,21,22,23,24,43,44,45,46,47,48,49,50,51,52,53,54,55,56,57,58,59,60]. Male predominance was noted (25 patients), with variable age at presentation (median 43 years), in 15 out of 30 patients (50%) with WBC > 10 × 10^9^/L. IRF2P2-RAR_α_ APL had different features at diagnosis (3 out 5 cases were female and all patients had WBC counts < 5 × 10^9^/L).

No CRs after ATRA or ATO-based regimens were observed among 2 patients with STAT3—RAR_α_ [54] or 1 with GTF2I-RAR_α_ [55].

ATRA monotherapy or combined with chemotherapy was the most frequent treatment for TBLR1-RAR_α_ APL, observing 2 out of 5 CRs and frequent relapse (including 1 extramedullary). The ATO-based regimen was successfully administered in 1 case at relapse [43,44,45,46].

Among STAT5B-RAR_α_ APL, treatment consisted of intensive chemotherapy plus ATRA (in most patients), with 8 out of 12 (67%) CRs and frequent relapse reported [7,15,16,17,18,19,20,21,22,23,24,49].

No resistances to ATRA plus chemotherapy induction were observed among patients with IRF2P2-RAR_α_, FIP1L1-RAR_α_, BCOR-RAR_α_, NuMa-RAR_α_, PRKAR1A-RAR_α_, FNDC3B-RAR_α_, TFG-RAR_α_, and NABP1-RAR_α_, but relapses were also frequent among these settings [47,48,49,50,51,52,53,56,57,58,59,60].

## 4. Discussion

This study shows that variant APL is probably less frequent (0.4%) than previously reported (1–2%). Our systematic literature search found 60 manuscripts on this topic (58 original reports and 2 reviews), almost equaling the number of patients with variant APL reported to date (*n* = 79). The characteristics of new 11 variant APL cases registered by the PETHEMA group are in line with prior reports (male predominance, PLZF-RAR_α_ as more frequent rearrangement). The vast majority of PETHEMA patients were treated with ATRA-containing schedules. While survival and CR rates seem better in our series as compared to background literature, prevention of relapse (especially in the CNS) remains as a challenging issue as well. Although prognosis of variant APLs seems better than non-promyelocytic AML, it is worse than PML-RAR_α_ APLs, where ATO + ATRA or AIDA-based regimens results in virtual absence of remission induction resistance, low rate of relapses (<15%), and higher rates of cure (>80%) [1].

As far as we know, this is the larger series analyzing the frequency of variant APL (0.4%); 11 out of 2895 patients with genetically confirmed APL had PLZF-RAR_α_ (0.3%) or NPM1-RAR_α_ (0.1%). This frequency is lower than 1.1% previously reported by Grimwade et al. [3], which showed 5 PLZF-RAR_α_ (0.8%) and 2 NPM1-RAR_α_ (0.3%) out of 611 APL. However, our frequency data should be carefully interpreted due to several reasons: (1) although the PETHEMA registry includes all suspected APL patients regardless of treatment or diagnosis, investigators may include preferably those cases evaluable for PML-RAR_α_ therapeutic protocols; (2) a number of registered APL patients were excluded from this study because of lack of genetic diagnosis. The majority of these cases had no genetic diagnosis because of very early death, but we cannot exclude that some of them had an unidentified variant APL; and (3) although the PETHEMA network offers expert counseling and possibility of genetic testing at central laboratories when required, the diagnosis of variant APL (in particular for very infrequent forms) needs first to be suspected and then guided PCR tests performed. In our opinion, the frequency of variant APL could be higher than herein reported, but probably lower than 1%.

The systematic literature review and the PETHEMA registry experience confirms some clinical features of variant APL that are common to PML-RAR_α_, as frequent coagulopathy and a median age at diagnosis of roughly 45 years old [64,65,66,67]. Interestingly, one out of two NPM1-RAR_α_ patients here reported was a pediatric patient, in line with prior case reports suggesting that this form is mainly affecting children (Table 3) [3,6,35,36,37,38,39,40,41]. As a distinction to PML-RAR_α_ APL, our study show that variant forms present with higher median WBC counts, and 40–50% could be classified as high-risk according to the Sanz’s score. As per the literature review, only IRF2P2-RAR_α_ APL had different features at diagnosis (3 out 5 cases were female and all had WBC < 5 × 10^9^/L) (Table 4) [7,15,16,17,18,19,20,21,22,23,24,43,44,45,46,47,48,49,50,51,52,53,54,55,56,57,58,59,60,61]. On the other hand, it has been suggested that CD56 could be frequently expressed in some variant APL forms [2,4], but this could not be confirmed in our series (CD56 available only in 5 patients).

Apart from accurate diagnosis, the main issue for the management of variant APL is the striking lack of evidence to guide therapeutic approaches in this population. Although our study aimed to shed light on this management, making reliable recommendations remains challenging in light of patient’s and therapeutic approaches heterogeneity reported so far. Thus, we can only make suggestions for induction therapy, as follows: (1) a chemotherapy-based approach could be administered for STAT3-RAR_α_ and GTF2I-RAR_α_ as 3 reported cases were resistant to ATRA- or ATO-based regimens; (2) an AIDA or AIDA-like induction could be employed for NPM1-RAR_α_, IRF2P2-RAR_α_, FIP1L1-RAR_α_, BCOR-RAR_α_, NuMa-RAR_α_, PRKAR1A-RAR_α_, FNDC3B-RAR_α_, TFG-RAR_α_, and NABP1-RAR_α_ (only 1 induction resistance reported). No clinical data regarding ATO sensitivity were available in the literature for these APL forms. However, in our series, one pediatric patient achieved a second CR after ATO plus ATRA plus intrathecal therapy for a concomitant bone marrow and CNS relapse, suggesting that ATO could be active as well in NPM1-RAR_α_ APL; (3) an ATRA plus chemotherapy-based regimen could be employed for TBLR1-RAR_α_ and STAT5B-RAR_α_, but the CR rate seems much lower than in PML-RAR_α_ APL. Of note, an ATO-based salvage was successfully used in 1 patient with relapsed TBLR1-RAR_α_ APL.

Regarding treatment and outcomes of PLZF-RAR_α_ APL, previously reported cases (*n* = 35) and data from our cohort of patients (*n* = 9) allow for more reliable recommendations. Based on previously published data, induction schedules were quite heterogeneous, including some ATO-based regimens, with low CR (34%) and high resistance rates (51%). Of note, better CR rates (64%) were observed among 14 patients receiving ATRA plus chemotherapy-based induction (Table 2). In contrast, front-line regimens were more homogeneous (AIDA or AIDA-like in 7 patients) in the PETHEMA cohort, no resistances occurred and early death was the only cause of induction failure, similar to PML-RAR_α_ patients [70]. Until we have better evidence, it seems reasonable to recommend a first induction with AIDA or AIDA-like regimens for PLZF-RAR_α_ patients. Regarding ATO therapy, although clinical responses are disappointing, combinations including ATO could play a role at relapse, where some responses have been observed so far (Table 1 and Table 2). The mechanism of resistance to ATRA and ATO in PLZF-RAR_α_ APL could be related to distinct nature of leukemogenic process as compared to the PML-RAR_α_. Rego et al., observed in vitro and in vivo, that therapeutic doses of ATRA and ATO can induce the degradation of both fusion protein, but maintenance of the leukemic phenotype depends on the continuous presence of the PML-RAR_α_ but not of the PLZF-RAR_α_ protein [71]. According to another study, ATRA, but not ATO, can provoke degradation of the PLZF-RARA fusion protein [27]. Moreover, it has been suggested that ATRA has difficulties to completely release the corepressor proteins like N-CoR (nuclear receptor corepressor) or nuclear receptor transcriptional (SMRT) from the PLZ-RAR_α_ fusion protein as there is a second binding site for corepressor complex in the PLZF region [72]. We should also highlight that PML restauration by ATO and ATRA activates p53 by recruiting the protein to PML-nuclear bodies site and promoting its activation. The lack of p53 function could be in relation to the resistance that is observed in APL variants [73]. On the other hand, activation of the hematopoietic growth factor granulocyte colony-stimulating factor (G-CSF) receptor signaling could lead to the release of corepressor proteins from PLZF, supporting the therapeutic combination of ATRA and G-CSF [12]. Other studies provided biological rationale to enhance the efficacy of ATRA through combinations with interferon [74] or histone deacetylase inhibitors [75,76] and efficacy of ATRA and ATO through 8-CPT-CAMP [77,78]. We should mention the possibility of using tamibarotene instead of ATRA for those patients with variants since it is thought that tamibarotene is a better inducer of differentiation and cells death in APL cells [79].

Post-remission outcomes were worse in variant APL as compared to classical forms, mainly due to frequent relapses. In the literature cases, crude relapse rate was 57% among PLZF-RAR_α_ and NPM1-RAR_α_, even if intensive consolidations were often administered. In our short series, 43% of patients relapsed with a remarkable frequency of CNS involvement, but a second CR was achieved in all patients. It should be noted that variant APLs frequently show risk factors that have been related with an increased risk of relapse in PML-RAR_α_ APL (i.e., hyperleukocytosis and male gender) [80]. In our series, 3 out of 4 relapses occurred in patients with more than 50 × 10^9^/L WBC counts at diagnosis. Given the high rate of relapses observed, we can speculate that consolidation regimens including ATRA and chemotherapy (i.e., anthracycline and Ara-C) may be useful to prevent hematological and extramedullary relapses. Regarding specific CNS prophylaxis, it is difficult to make recommendations given the low number of cases, but it seems judicious to follow similar approaches than for typical APL. Although our short but mature series shows an acceptable proportion of patients still alive (73%), better than in previously published data. We can hypothesize that more homogeneous schedules with ATRA plus chemotherapy-based regimens, successful salvages, and more contemporaneous treatments, could explain our survival results. From our experience, the role of alloHSCT in first CR is debatable, but it should be performed when possible in second CR.

In conclusion, we confirm that variant forms are very rare, accounting for less than 1% of APLs. Main characteristics of 11 patients reported by PETHEMA are in line with previous reports (male predominance, high WBC, median age 45 years, PLZF-RAR_α_ followed by NPM1-RAR_α_ as more frequent rearrangements). Except for STAT3-RAR_α_ and GTF2I-RAR_α_ APLs, ATRA plus chemotherapy-based induction may lead to high CR rates. Further studies are needed to gain insights on optimal post-remission strategies to prevent the relatively high rate of relapse observed in variant APL patients.

## Figures and Tables

**Table 1:** Clinical characteristics, treatment, and outcomes of 11 variant acute promyelocytic leukemia (APL) patients reported to the PETHEMA registry.

Sex/Age (Years)	WBC × 10^9^/L	Therapy-Related APL	Coagulo-Pathy	CD56 %	Karyotype & Rearrangement by PCR	Front-Line Therapy	Induction Response	Relapse	Salvage Therapy	AlloHSCT (Type)	Survival in Months (Status)
**PLZF-RAR_α_ patients**											
M/31	NA	No	NA	NA	t(11;17)(q23;q21) PLZF-RAR_α_	**Induction** AIDA (PETHEMA 99), **Consolidation**, **Maintenance** 6-MP/MTX/ATRA	CR	YES (isolated CNS sarcoma) at 58 months	**ATRA + ATO + TIT—CR2, RTH**, consolidation HD-Ara-C + IDA; maintenance 6-MP/MTX	NO	101+
F/50	2.9	Yes: MTX for arthritis	YES	100	t(11;17)(q23;q21) PLZF-RAR_α_	**Induction** ATRA + IDICE, **Consolidation** Ara-C/MTZ/ATRA (×2), **alloHSCT**	CR	NO	-	YES (MUD)	36+
M/76	21.4	Yes: chemotherapy for colon cancer	YES	NA	t(11;17)(q23;q21) PLZF-RAR_α_	**Induction** AIDA (PETHEMA2012)	Death	-	-	-	0.3 (death)
M/67	53	No	YES	47	t(11;17)(q23;q21) PLZF-RAR_α_	**Induction** AIDA (PETHEMA 2012), **Consolidations** 1st: IDA/Ara-C/ATRA, 2nd:MTZ/ATRA, 3rd: IDA/Ara-C/ATRA **Maintenance** 6-MP/MTX/ATRA (2 years)	CR	YES (bone marrow) 1st at 40 months 2nd at 52 months 3rd at 60 months	**ATRA + ATO—CR2** **2nd relapse**: MTZ + Ara-C—**CR3** consolidation Mylotarg (×2) **3rd relapse:** untreated	NO	60 (death)
M/43	16.8	No	YES	0	t(11;17)(q23;q21) PLZF-RAR_α_	**Induction** AIDA (PETHEMA 2012)	Death	-	-	-	0.2 (death)
M/40	9.6	No	YES	0	t(11;17)(q23;q21) PLZF-RAR_α_	**Induction** ATRA + IA 3+7, **Consolidation** HD-Ara-C (×1), alloHSCT	CR	NO		YES (MUD)	47+
M/3 months	56.4	No	YES	NA	t(11;17)(q23;q21) PLZF-RAR_α_	**Induction** ATRA + AIE + ithAra-C, **Consolidation** ATRA + AI + ithAra-C (×1) ATRA + HAM + ithAra-C (×1), **Intensification**: ATRA + HAE + ithAra-C, **Maintenance**: ATRA + 6-TG Ara-C s.c. + ithAra-C + RTH CNS	CR	NO	-	NO	48+
M/33	248	No	NA	NA	t(11;17)(q23;q21) PLZF-RAR_α_	**Induction** DA 3+7, **Consolidation** DA 3+7	CR	YES (isolated CNS) 1st at 24 months 2nd at 34 months 3rd at 50 months	**HD-Ara-C + TIT (×8)—CR2**, alloHSCT **2nd relapse:** Ara-C + MTZ + TIT (×7) plus radiotherapy—**CR3** **3rd relapse:** ATRA + FLAG-IDA—CR4 consolidation ATRA + ATO	YES (MRD)	62+
M/62	1.1	No	NA	0	t(11;17)(q23;q21) PLZF-RAR_α_	**Induction** IA 3+7 (×2), **Consolidation** HD-Ara-C (×2)	CR	NO	-	NO	8+
**NPM1-RAR_α_ patients**											
F/7	100	No	NA	NA	t(5;15;17) NPM1-RAR_α_	**Induction** AIDA (PETHEMA 2017), **Consolidation**: 1st: IDA/Ara-C/ATRA, 2nd: MTZ/ATRA, 3rd: IDA/Ara-C/ATRA	CR	YES (CNS + bone marrow) at 9 months	**ATRA + ATO + TIT—CR2**, alloHSCT planned	planned (haplo-identical)	11+
M/74	1.7	No	NO	0	t(5;17)(q35;q21) NPM1-RAR_α_	**Induction** FLUGA → RES 2nd line AIDA → PR 3rd line IA 2+5 → CR, **Consolidation** intermediate-dose Ara-C (×2)	RES	NO	-	NO	24+

Abbreviations: M: male, F: female, NA: not available, CR: complete response, WBC: leukocytes, APL: acute promyelocytic leukemia, PCR: polymerase chain reaction, RES: resistance, PR: partial response, PLZF: promyelocytic leukaemia zinc finger, PETHEMA: Programa para el Tratamiento de Hemopatias Malignas, MTX: methotrexate, MRD: matched related donor, alloHSCT: allogeneic hematopoietic stem cell transplantation, MUD: matched unrelated donor, CNS: central nervous system, TIT: triple intrathecal therapy, ith: intratheca:, RTH: radiotherapy, s.c.: subcutaneous, ATRA: all-trans-retinoic acid, IDA: idarubicin, 6-MP: 6 mercaptopurine, 6-TG: 6- thioguanine, MTX: methotrexate, Ara-C: citarabine, HD-Ara-C: high dose Ara-C, MTZ: Mitoxantrone, ATO: arsenic trioxide, AIDA (IDA12 mg/m^2^ days 2, 4, 6, and 8, ATRA 45 mg/m^2^ till CR), IDICE (IDA 12 mg/m^2^ day 1, 3 and 5, Ara-C 500 mg/m^2^/12 h days 1, 3, 5, and 7, Etoposide 100 mg/m^2^ days 1, 2 and 3), DA 3+7 (Daunorubicin 60 mg/m^2^ days 1–3, Ara-C 200 mg/m^2^/12 h days 1–7), IA 3+7 (IDA 12 mg/m^2^ days 1–3, Ara-C 200 mg/m^2^/12 h days 1–7), AML-BFM 2004 INTERIM: induction AIE (Ara-C 100 mg/m^2^/24 h days 1–2, Ara-C 100 mg/m^2^/12 h days 3–8, Ida 12 mg/m^2^ days 3,5 and 7, Etoposide 150 mg/m^2^ days 6–8) plus ith Ara-C days 1 and 8, consolidation AI (Ara-C 500 mg/m^2^/24 h days 1–4, Ida 7 mg/m^2^ days 3, 5) plus ith Ara-C days 0 and 6, consolidation HAM (HD-Ara-C 1 g/m^2^/12 h days 1–3, MTZ 10 mg/m^2^ days 3–4) plus ith Ara-C days 0 and 6, intensification HAE (HD-Ara-C 3 g/m^2^/12 h days 1–3, ETO 125 mg/m^2^, days 2, 3, 4, 5) plus ith Ara-C day 0, maintenance (6-TG 40 mg/ m^2^ daily, Ara-C 40 mg/m^2^ s.c., for 4 days, every 4 weeks) plus ith Ara-C and RTH CNS 15 Gy, HD-Ara-C and ith Ara-C doses are modified according to age. FLUGA (Fludarabine 25 mg/m²/d i.v. days 26–, Ara-C 75 mg/m²/d i.v. days 2–5, g-CSF 5 µgr/kg/d s.c. days 1–3), FLAG-IDA (fludarabine 30 mg/m^2^, AraC 2 g/m^2^ for 5 days, idarubicin 10 mg/m^2^ for 3 days, and G-CSF 5 micro g/kg from day +6 until neutrophil recovery).

**Table 2:** Characteristics and clinical outcomes in patients reported with PLZF-RARa.

Cases/First Reference	Karyotype	Sex/Age (Years)	WBC (×10^9^/L)	Response to ATRA-Containing Regimen	Response to ATO-Containing Regimen	Induction(s) and Response(s)	Relapse (Months) Salvage Therapy	AlloHSCT	Survival in Months (Status)
**ATRA monotherapy induction**									
[63]	46,XY,t(11;17)(q23;q21)	M/67	4.1	Differentiation	-	**Induction** ATRA—**ED**	-	-	0.7 (death)
[11]	46,XY,t(11;17)(q23;q21)	F/81	7.6	Differentiation	-	**Induction** ATRA—**ED**	-	-	0.6 (death, cerebral bleeding)
	46,XY,t(11;17)(q23;q21)	M/67	4.1	Not evaluable	-	**Induction** ATRA—**ED**	-	-	0.5 (death)
[7]	46,XX,t(11;17)(q23;q21);47,idem,+22	F/48	42.5	Not evaluable	-	**Induction** ATRA—**ED**	-	-	0.3 (death by cerebral bleeding)
[34]	46,XY, t(11;17)(q23;q21) with del(5)(q22q35)	M/53	15.4	NO	-	**Induction** ATRA—**RES**, DNR + Ara-C—**CR**	NA	NA	NA
**ATRA + chemotherapy-based induction**									
[12]	46,XY,t(11;17)(q23;q21)	M/31	69.5	YES	-	**Induction** ATRA + Ara-C + IDA (3+7)—**CR**, **Consolidation** Ara-C + amsacrine, ETO + MTZ	YES (11) ATRA—**CR2**, consolidation HD-Ara-C, **alloHSCT**	YES in CR2	51+
[13]	46,XY,t(11;17)(q23;q21)	M/83	NA	YES	-	**Induction**: ATRA + DNR—**CR Consolidation** DNR + Ara-C × 2, **Maintenance** ATRA +6-MP + MTX	NO	NO	24+
[49]	47,XY,+8/47,XY,+8,t(11;17)(q23;q21)	M/62	1.2	YES	-	**Induction** ATRA + ADE—**CR**	YES (7) NA	NO	17+
[10]	46,XY,t(11;17)(q23;q21)/46,XY	M/53	4.5	YES	-	**Induction** ATRA + ADE—**CR**, **Consolidation** ADE, MACE, MiDAC	YES (45) FLAG × 2 + ATRA—**CR2, alloHSCT**	YES in CR2	177+
[26]	46,XY,t(7;17)(q35;q21)	M/58	7.4	YES	-	**Induction** ATRA + DAT—**CR**, **Consolidation** DAT + ATRA, MACE	YES (36) NA	NO	36 (death in relapse)
[8]	46,XY,t(11;17)(q23;q21)/45,X,-Y,t(11;17)(q23;q21)	M/50	6.8	YES	-	**Induction** ATRA + ADE—**CR**, **Consolidation** ADE, MACE, MiDAC	NO	NO	73+
[3]	46,XY, t(11;17)(q23;q21)/46,idem,del(12)(p1?)/46,idem,-6,+r	M/75	2.0	YES	-	**Induction** ATRA + DAT—**CR**, **Consolidation** DAT, MACE	YES (55) DNR + Ara-C—**CR2**	NO	88 (death in CR2)
[3]	45,X,2Y,t(11;17)(q23;q21)	M/32	11.6	YES	-	**Induction** AIDA—**CR, alloHSCT**	NO	YES in CR1	37+
	46,XY,i(7)(q10),t(11;17)(q23;q21)	M/43	10.4	YES	-	**Induction** AIDA—**CR**	YES (30) NO	NO	30 (death in relapse)
[28]	46,XY,t(11;17)(q23;q21)/47,idem,+8	M/68	6.9	Differentiation (monotherapy in second line)	-	**Induction**: ATRA + DNR + Ara-C—**RES**, ATRA (monotherapy)—**RES**, MTZ + HD-Ara-C—CR	YES (15) NO	NO	15 (death in relapse)
[11]	45,X,2Y,add(2)(q33),t(11;17)(q23;q21)/46,XY	M/34	2.4	NO	-	**Induction** ATRA + DNR + Ara-C—**RES**, Amsacrine + HD-Ara-C—**CR**	YES (56) NO	NO	57 (death in relapse)
[14]	No metaphases	M/48	71.6	NO	NO (in second line)	**Induction** ATRA + DNR × 2—**RES**, HIDAC + ATO—**RES**	-	-	NA
[14]	46,XX,t(11;17)(q23;q22)	F/38	23.6	NO	-	**Induction** ATRA + DNR—**RES**, MTZ + ETO + Ara-C—**ED**	-	-	2 (death by sepsis)
[32]	46,XX,add(17)(q21)[4]/46,XX[9]. ish der(11)t(11;17)(q23;q21)	M/81	1.8	Not evaluable	-	**Induction** AIDA—**ED**	-	-	0.3 (death by pulmonary bleeding)
**Chemotherapy-based induction**									
[11]	46XY+(3)+(13)(q34), t(11;17)(q23;q21)	M/53	15.3	-	-	**Induction** DNR + Ara-C—**CR** **Consolidation** DNR + Ara-C	YES (7) DNR + Ara-C + Gm-CSF—**CR2** 2nd relapse (19) ETO + MTZ—**CR3** ATRA + other—**RES** 3nd relapse (23)	NO	28 (death in relapse)
[31]	t(11;17)(q23;q21)	M/52	1.6	-	-	**Induction** chemotherapy—**CR**	NA	NA	NA
[29]	46,XY,t(11;17)(q23;q21)	M/37	45.2	-	-	**Induction** DNR + Ara-C—**RES**, IFN-alpha—**RES**, ETO + MTZ—**CR**	NO	NO	11 (death cause unknown)
[3]	46,XY,t(11;17)(q23;q21),idem,-Y/46,XY	M/34	20	YES (in second line)	-	**Induction** DNR + Ara-C + ETO—**RES**, Ara-C + IDA+ATRA—**CR, alloHSCT**	NO	YES in CR1	33+
[33]	t(11;17)(q23;q21)	M/50	1.3	YES (in second line)	YES (in second line)	**Induction** DNR + Ara-C—**RES**, ATRA + ATO—**CR**	NA	NA	NA
[3]	46,XY.ish,ins(11;17)(q23;q21,q21)	M/62	9.9	YES (monotherapy at relapse)	-	**Induction** Ida + Ara-C + ETO—**RES**, MICE—**CR**	YES ATRA-**CR2** 2nd relapse (8)—no treatment	NO	25 (death in relapse)
**ATO+ATRA+/-chemotherapy-based induction**									
[7]	46,XX,der(11),der(17)/46,XX	F/60	34	YES	YES	**Induction** ATRA + ATO + IDA—**CR**, **Consolidation** ATO + IA, MTZ, ATO + DNR, ATO + DA, MA	NO	NO	11 +
	46,XY,?t(11;17)(q23;q21)/46,XY	M/44	52.1	NO	NO	**Induction** ATRA + DNR + Ara-C (3+7) + ATO—**RES**	-	-	5+
	47,XY,+8[4/20]/47,idem,t(11;17)(q23;q21)/46,XY	M/52	8.9	YES (in second line)	NO	**Induction** ATRA+ATO—**RES**, DNR + ATRA + CAG × 3—**CR**	NO	NO	7+
	46,XX,t(11;17)(q23;q22)/46,XY	M/46	23.1	NO	NO	**Induction** ATRA + ATO—**RES**, IDA + Ara-C—**CR**, **Consolidation** MTZ + ETO + Ara-C, MTZ + Ara-C, HD-AraC	NO	NO	5+
**ATO+/-chemotherapy-based induction**									
[9]	45,X,-Y, t(11;17)(q23;q21)/46,XY, t(11;17)(q23;q21)	M/23	9.1	-	NO	**Induction** ATO—**RES**, DNR + Ara-C 3+7—**CR**; **Consolidation** Ara-C × 5, Ara-C + DNR × 4	YES (7) NA	NO	32+
[30]	NA	M/15	64.9	-	NO	**Induction** ATO + IDA—**RES**, HD-Ara-C (×3)—**CR**, **Maintenance** ATRA	NO	NO	NA
	NA	M/38	NA	-	NO	**Induction** ATO—**RES**	-	-	2+
	NA	M/45	NA	-	NO	**Induction** ATO—**RES**	-	-	NA
	NA	M/36	4.9	-	NO	**Induction** ATO + Decitabine—**RES**, DNR + Ara-C—**RES**, HD-Ara-C—**CR, Consolidation** HD-Ara-C × 2	YES (11) NO	NO	11 (death in relapse)
	NA	M/22	76.9	-	NO	**Induction** ATO—**RES**, HD-Ara-C (×3)—**NA**	NA	NA	NA

Abbreviations: M: male, F: female, NA: not available, WBC: leukocytes, CR: complete response, RES: resistance, ED: early death, alloHSCT: allogeneic hematopoietic stem cell transplantation, ATRA: all-trans-retinoic acid, ATO: arsenic trioxide, G-CSF: Granulocyte colony-stimulating factor, DNR: daunorubicine, IDA: idarubicin, Ara-C: cytarabine, HD-Ara-C: high dose Ara-C, IFN: interpherone, ETO: etoposide, MTZ: Mitoxantrone, MTX: mitoxantrone, 6-MP: 6-mercaptopurinum, MACE (Amsacrine, Ara-C, Etoposide), MiDAC (mitoxantrone, Ara-C), IA (Ida plus Ara-C), FLAG (fludarabine, Ara-C, G-CSF), MA (Mitoxantrone, Ara-C), CAG (Ara-C, aclarubin and G-CSF), MICE (Ara-C, Etoposide, Gemtuzumab ozogamicin, Ida, Mitoxantrone), AIDA (ATRA plus IDA), ADE (Ara-C, ADR plus ETO), DAT (DNR, Ara-C plus thioguanine), DA (DNR plus Ara-C)

**Table 3:** Characteristics, treatment, and outcomes of 9 NPM1-RARa APL patients reported in the literature.

Case Reference	Karyotype	Sex/Age (Years)	WBC (×10^9^/L)	Myeloid Sarcoma	Response to ATRA-Containing Regimen	Response to ATO-Containing Regimen	Induction and Response	Relapse (Months) Salvage Therapy	alloHSCT	Survival in Months (Status)
[37,40]	46,XX,t(5;17)(q32;q12) 48,XX,t(5;17),+2mar	F/2	NA	NO	YES	-	**Induction** DNR + Ara-C + ETO—**CR**, then ATRA—**molecular CR,** autoHS**CT**	YES (7)	NO (autoHSCT)	7 (death in relapse)
[41]	47,XY,t(5;17)(q35;q21),der(8)(p23), der(10)(q26),del(12)(q13q22),del(1)(q12q14),−16,−18,+21,+22,+mar	M/12	NA	NO	YES	-	**Induction** DNR + Ara-C (3+7)—**CR**, consolidation DNR + Ara-C + 6-TG + ETO + DXM	YES (5) ATRA + Ara-C—**CR2**, alloHSCT	YES in CR2	8+
[3]	46,XX,ins(3;5)(q26;q13q13),t(5;17)(q34;q21)	F/9	17	NO	YES	-	**Induction** ATRA + Ara-C + MTZ—**CR**, consolidation DNR + Ara-C + ETO	NO	NO	29+
	46,XX,der(5)t(5;17)(q13;q21),del(8)(q22q24),der(17),532–dim	F/76	43.1	NO	YES	-	**Induction** ATRA + DNR + Ara-C—**ED**	-	-	0.5 (death by differentiation syndrome)
[6]	46,XY,t(5;17)(q35;q21),del(12)(p13)	M/12	15.7	NO	Not evaluable	-	**Induction** ATRA—**ED**	-	-	0.2 (death by cerebral hemorrhage)
[39]	46,XY,t(5;17)(q35;q21)	M/29	2.9	Pelvis	YES	NA (used in relapse)	**Induction** AIDA—**CR** consolidation IDA + Ara- + ATRA), MTZ + ETO + ATRA, IDA + Ara-C + 6-TG + ATRA, maintenance ATRA	YES (22) ATO (1 cycle)	NO	23+
[36]	46,XY,t(5;17)(q35;q12)[4]/46,XY[16]	M/4	NA	Skin	YES	-	**Induction** ATRA + chemotherapy—**CR**	YES (NA)	NO	46+
[38]	46,XY,t(5;17)(q35;q12)	M/51	4.2	Vertebral	YES	-	**Induction** ATRA + chemotherapy—**CR**	NO	NO	2+
[35]	46,XY,t(5;17)(q35;q12)	M/64	NA	No	Yes	-	**Induction** ATRA + chemotherapy—**CR**	NO	NO	18+

Abbreviations: Ref: references, M: male, F: female, NA: not available, CR: complete response, ED: early death, alloHSCT: allogeneic hematopoietic stem cell transplantation, autoHSCT: autologous hematopoietic stem cell transplantation, DIC: disseminated intravascular coagulopathy, ATRA: all-trans-retinoic acid, ATO: arsenic trioxide, Ara-C: cytarabine, DNR: daunorubicine, IDA: idarubicin, 6-TG: 6-thioguanine, ETO: etoposide, DXM: dexamethasone, MTZ: Mitoxantrone.

**Table 4:** Cases reported in the literature with other variant APL.

References	Rearrangement	Karyotype	N of Cases	Sex/Median Age Years (Range)	WBC × 10^9^/L Median (Range)	ATRA Sensitivity	ATO Sensitivity	Treatment Schedules	Relapse Months	HSCT	OS (Months)
[8,15,16,17,18,19,20,21,22,23,24]	STAT5B-RAR_α_	From normal till complex karyotype, (interstitial deletion within chr. 17)	12	M (10)/F (3) 42 (17–67) years	16.1 (2.1–77.8)	YES	POSSIBLE 1 CR with ATRA + ATO + Ida	IA, ETO, FLAG, CAG, FLA, DA, MTZ, GO, Decitabine, ATO (most of them with ATRA)—**CR in 8/12 (67%)** cases (5 CR with ATRA-containing induction)	YES (4 patients) Median 30.4, range 3.5–56	YES 2 in CR1 and 2 in CR2	Median 25.6 (0–75)
[43,44,45,46]	TBLR1-RAR_α_ (1 case also with PML-RAR_α_)	t(3;17)(q26;q12–21) plus other alterations t(3;17) (p25;q21) in 1 case	6	M (6)/F (2) 41.3 (3–72) years (available in 4 patients)	14.1 and 20.4 (available)	YES	POSSIBLE (in 2nd line with chemotherapy)	1st case: ATRA + DNR—**CR**, 2nd case: ATRA + MTZ—**RES**, 3rd case: ATRA + chemotherapy—**ED** 4th case: ATRA—**RES** 5th case: ATRA—**RES**, chemotherapy **CR** 6th case: —**ED**	YES (2 patients after 10 and 24 months; one extramedullary)	YES (1 patient in 2nd CR—cord blood)	1 patient alive after alloHSCT
[47,48,49,50,51]	IRF2P2-RAR_α_	Normal, diploid, −X (2 cases), t(1;17)(q42;q21)	5	M(2)/F (3) 38 (19–68) years	3.8 (1.65–5.14)	YES	YES	1st case: ATRA + ATO + GO—**CR**/2nd case: ATRA + IDA + Ara-C + GO—**CR** 3rd case: ATRA—**CR** 4th case: ATRA + IDA + Ara-C—**CR** 5th case: ATRA + ATO + DRN + Ara-C—**CR**	YES (3 patients between 8–12 months)	1 patient (after relapse)	Median 39 (8–18) months
[52,53]	FIP1L1-RAR_α_	t(4;17)(q12;q21)	2	F (2) 77, 90	59 in 1 case (other case NA)	YES	-	1st case:**Induction** AIDA protocol—**ED**; 2nd case: **Induction** ATRA—**CR**	NA	NO	1st patient: 0.3 (death by DS), 2nd patient NA
[54]	STAT3-RAR_α_	45,XY,-Y[6]/46,XY[8] or 46,XY[20]	2	M (2) 24, 26	6.6, 32.3	NO	NO	1st case: **Induction** ATO + ATRA—**RES**, DA3+7—**RES**, Homoharringtonine + ARA-C + G-CSF—**CR**, consolidation FLU + ARA-C (×4) MTZ + ARA-C (×1) 2nd case: **Induction** ATRA—**RES**, IA3+7—**RES**	YES (1st patient)	NO	1st patient: 33 (death in relapse), 2nd patient 6 months (death as RES)
[55,58]	BCOR-RAR_α_	t(X;17)(p11;q21) or Y,t(X;17)(p11.4;q21)	2	M/45 and 71	25.3 and >10	YES	NO (in relapse)	1st case:**Induction** ATRA + IDA + Ara-C—**CR**, than **Consolidation** 2nd case: **Induction** DA 3+7—**CR**, **Consolidation** ATRA + chemotherapy	YES at 35 months IDA + Ara-C + ATRA—**CR2** 2nd at 41 months—ATO—**RES**, tamibarotene + DNR + Ara-C—**CR3**	YES (cord blood in 3rd CR)	44+ and 12+
[56]	GTF2I-RAR_α_	t(7;17)(q11;q21)	1	M/35	53.7	No	NO	**Induction** ATRA + DA - **RES** ID-ARA-C – **RES** IAH – **RES** ATRA+ATO – **RES**, death	-	-	4.8 (death as RES)
[57]	NuMa1-RAR_α_	t(11;17)(q13;q21)	1	M/0.5	3.6	YES	-	**Induction** ATRA—**CR**, autoHSCT	NO	NO (autoHSCT)	38
[59]	PRKAR1A-RAR_α_	t(17;17)(q21;q24)	1	M/66	5.3	YES	YES	**Induction** ATRA + ATO + IDA—**CR**, Consolidation Ara-C + amsacrine × 3, Maintenance ATRA	NO	NO	24+
[60]	NABP1-RAR_α_ (OBFC2A-RAR_α_)	der(2)(t(2;17)(q32;q21) with sublcones t(11;19)(q13;p13.1)	1	M/59	96.9	YES	-	**Induction** AIDA—**CR** Consolidation × 2 cycles	NO	YES (MUD)	15+
[62]	TFG-RAR_α_	t(3;14;17)(q12;q11;q21)	1	M/16	1.81	YES		**Induction**: ATRA—**CR, Consolidation**: ATRA + IDA (×2), **maintenance**: ATRA	NO	NO	NA
[61]	FNDC3B-RAR_α_	t(3;17)(q26;q21)	1	M/36	3.6	YES	-	**Induction** ATRA + DA 3+7—**CR**, consolidation DA 5+2, HD-Ara-C, maintenance ATRA/MTX/6-MP	YES	NO	8 (death in relapse)

Ref: references, N: number, M: male, F: female, WBC: leukocytes, CR: complete response, RES: resistance, OS: overall survival, ED: early death, HSCT: hematopoietic stem cell transplantation, allo: allogenic, auto: autologous, DS: differentiation syndrome, ATRA: all-trans-retinoic acid, ATO: arsenic trioxide, GO: gentuzumab ozogamizine; ETO: etoposide, IDA: idarubicine, DNR: daunorubicine, G-CSF: Granulocyte colony-stimulating factor, MTZ: mitoxantrone, FLU: fludarabine, MTX: metotrexate, MTZ: mitoxantrone, 6-MP: 6-mercaptopurine, ID-Ara-C: intermediate dose Ara-C, IA/IA 3+7: Ida plus Ara-C, DA: DNR plus Ara-C, FLA: fludarabine plus Ara-C, FLAG: fludarabine, Ara-C, G-CSF, CAG: Ara-C, aclarubin, G-CSF, AIDA: ATRA plus IDA, IAH: Ida plus Ara-C plus homoharringtonine.
